# Ectoparasites of the European wildcat (*Felis silvestris*) in Germany

**DOI:** 10.1016/j.ijppaw.2024.100977

**Published:** 2024-08-24

**Authors:** Katrin Bisterfeld, Marie-Kristin Raulf, Andrea Springer, Johannes Lang, Michael Lierz, Christina Strube, Ursula Siebert

**Affiliations:** a Institute for Parasitology, Centre for Infection Medicine, University of Veterinary Medicine Hannover, Buenteweg 17, 30559 Hanover, Germany; b Institute for Terrestrial and Aquatic Wildlife Research, University of Veterinary Medicine Hannover, Werftstrasse 6, 25761 Buesum, Germany; c Clinic for Birds, Reptiles, Amphibians and Fish, Justus-Liebig-University Giessen, Frankfurter Strasse 114, 35392 Giessen, Germany

**Keywords:** Ticks, Fleas, Mites, Lice, Prevalence, Epidemiology

## Abstract

Understanding the impact of parasites on wildlife populations is an important aspect of conservation management. However, research on ectoparasites in wildlife can be difficult, as examinations of live animals which are not habituated to human handling are often impossible. The European wildcat (*Felis silvestris*) is a strictly protected wildlife species whose population has been recovering in Germany in recent decades. Several studies from different European countries have investigated the parasitological status of European wildcat populations. However, most of these studies assessed endoparasite infections, whereas ectoparasite infestations have often been neglected. To fill this knowledge gap for wildcats in Germany, 131 dead found specimens were examined for ectoparasites by macroscopic and microscopic examination of the fur and the ear canals. Infestation with ectoparasites was present in 84.0% (110/131) of the wildcats. Ticks showed the highest prevalence with 72.5% (95/131) of wildcats infested, with 49.6% (65/131) infested with *Ixodes ricinus* and 36.6% (48/131) with *Ixodes hexagonus*/*canisuga*. A total of 27.5% (36/131) of the wildcats were positive for at least one flea species. Of the nine different flea species identified by morphology and/or molecular analyses, Ceratophyllidae were most common (16.8% [22/131]), with *Ceratophyllus sciurorum* confirmed on 12.2% (16/131) and *Nosopsyllus fasciatus* on 1.5% (2/131) animals, followed by *Pulex irritans* (5.3% [7/131]), *Spilopsyllus cuniculi* (3.8% [5/131]), *Chaetopsylla* spp. (3.1% [4/131]) (2/131 *Chaetopsylla trichosa* and 1/131 *Chaetopsylla globiceps*), *Ctenocephalides felis* (1.5% [2/131]), *Archaeopsylla erinacei* (1.5% [2/131]) and *Ctenophthalmus baeticus* (0.8% [1/131]). Further, 23.7% (31/131) of the wildcats harboured mites, identified as *Trombicula autumnalis* in 12.2% (16/131) and *Otodectes cynotis* in 4.8% (6/124) of cases. The only louse species detected was *Felicola hercynianus* with a prevalence of 2.3% (3/131). Infestation intensities ranged from 1 to 86 ticks, 1–49 fleas, 1–1896 mites, and 1–92 *F. hercynianus* per wildcat. This study demonstrates that a variety of ectoparasites infests wildcats in Germany, but they do not seem to have a serious impact on the general health of wildcats, as judged by the hosts' mostly good or very good nutritional condition. In addition, the potential risk to domestic cats (*Felis catus*) and humans posed by the wildcats’ ectoparasites, appears to be low but present.

## Highlights

• Ectoparasite infestation was present in 84.0% of 131 dead-found European wildcats.
• A total of 15 ectoparasite species were identified morphologically and molecularly.
• Ticks showed the highest prevalence, with *Ixodes ricinus* as the most frequent species.
• Nine flea, two mite and one louse (*Felicola hercynianus*) species were detected.
• General health impact seems to be low considering the cats' good nutritional condition.

## Graphical abstract

**Figure:**
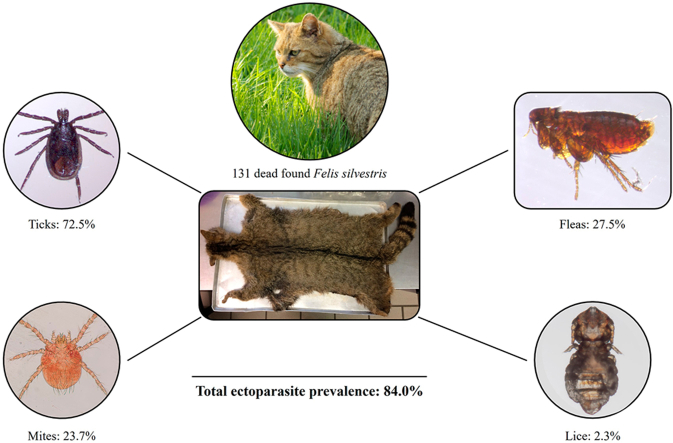

## 1 Introduction

Parasitism in wildlife is common, whereby the parasite benefits most when the host is weakened but not immobilised or killed, so that effective reproduction and spread of the parasite can take place (Ewald, 1983). However, if such an optimal virulence level is not maintained, excessive ectoparasite infestation may have detrimental effects on a population's health, either directly by causing e.g. dermatitis, inflammatory skin reactions, secondary bacterial infections, and anaemia, or indirectly via transmission of vector-borne pathogens (Deplazes et al., 2020; Wall and Shearer, 2008).

The European wildcat (*Felis silvestris*; hereafter wildcat) population was close to being eradicated mainly from hunting and trapping in the early 20th century and habitat fragmentation in the late 20th century, after it was widespread throughout Germany in former times (Piechocki, 1990). Today, wildcats are considered as Threatened according to the German Red List (Meinig et al., 2020) and are still strictly protected (Bundesnaturschutzgesetz, 2009; EEC, 1992). Due to various species conservation projects, e.g. those of the German Federation for the Environment and Nature Conservation, Friends of the Earth Germany (*Bund fuer Umwelt und Naturschutz Deutschland*, *BUND*), the population's recovery in central and south western parts of Germany was promoted, with an estimated population size of 5000 to 7000 individuals in 2017 (Balzer et al., 2018).

In addition to potential harm from ectoparasites, wild animals, such as wildcats*,* may serve as natural pathogen reservoirs and can be a source of spillover events to domestic animals and humans (Thompson et al., 2010). Therefore, parasite surveillance is required to evaluate the necessity for further conservation measures and to identify possible health risks for domestic animals in terms of the One Health approach. So far, little is known about the relevance of ectoparasite infestation of the wildcat.

Ticks are ectoparasites that infest wild and domestic animals and humans all over the world (Estrada-Peña et al., 2018). *Ixodes ricinus* is the most common tick species in Europe and infests about 200 different vertebrate species (Deplazes et al., 2020; Estrada-Peña et al., 2018). Other species, particularly *Ixodes hexagonus* and *Ixodes canisuga,* are also frequently found on European wildlife such as hedgehogs (*Erinaceus europaeus*), red foxes (*Vulpes*), polecats (*Mustela putorius*), and badgers (*Meles meles*) (Deplazes et al., 2020; Estrada-Peña et al., 2018). *Ixodes* ticks, including *I. ricinus* and *I. hexagonus,* have been found on wildcats, e.g. in Germany, France, and Spain (Dominguez, 2004; Leple, 2001; Steeb, 2015). In addition to ticks, fleas are common ectoparasites of wildcats (Piechocki, 1990). Piechocki (1990) observed the cat flea *Ctenocephalides felis* on wildcats, and infestations with the rabbit flea *Spilopsyllus cuniculi,* which he attributed to the high encounter rates of rabbits (*Oryctolagus cuniculus*) with wildcats (Piechocki, 1990). In Spain, *C. felis*, *S. cuniculi*, *Ceratophyllus sciurorum*, *Ctenophthalmus* spp., and *Peromyscopsylla spectabilis spectabilis* have been detected on wildcats (Dominguez, 2004). Several mite species may infest felids, including *Cheyletiella blakei*, *Demodex cati*, and *Demodex gatoi*, *Trombicula autumnalis*, *Otodectes cynotis*, and *Notoedres cati* (Bowman et al., 2002; Deplazes et al., 2020), and Cheyletiellidae, Sarcoptidae, and *Demodex* spp. have already been detected on wildcats (Leple, 2001; Steeb, 2015). Sarcoptic mange has been known in foxes in Scandinavia since the 1970s (Davidson et al., 2008), whereas the first detection of *Sarcoptes scabiei* in a wildcat in Spain was in 2021 (Najera et al., 2021). The chewing louse *Felicola hercynianus* is specifically attributed to wildcats and is morphologically distinct from that of the domestic cat (*Felis catus*), *Felicola subrostratus*. Apart from two cases in Scotland (Kéler, 1957), infestations of wildcats with *F. subrostratus* have not been described (Martín Mateo, 2009; Mey, 1988).

The aim of this study was to provide an overview on ectoparasite species, their prevalence and infestation intensity in wildcats in Germany, to aid in elucidating whether wildcats could serve as an ectoparasite reservoir for domestic cats with potential risks of spillover events.

## 2 Materials and methods

### 2.1 Origin of wildcat samples

**Fig. 1:**
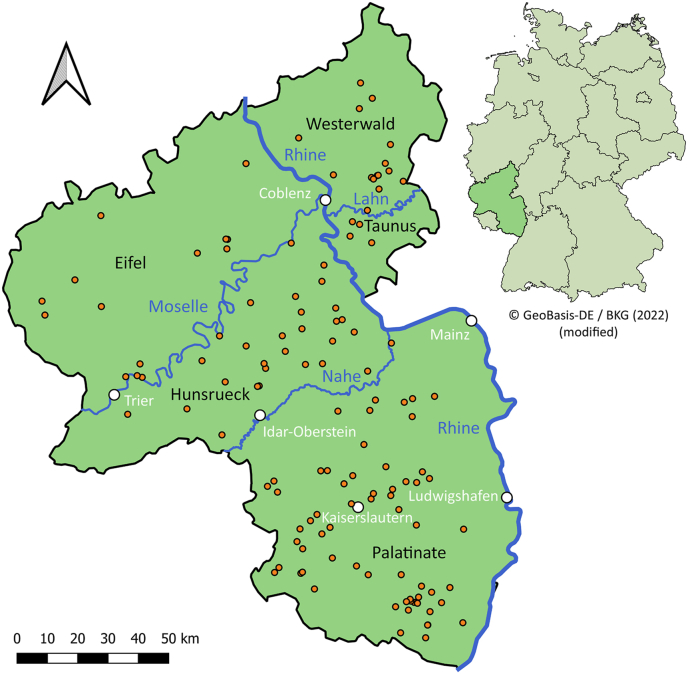
Map of the German state Rhineland-Palatinate showing the sampling locations of dead found wildcats (orange dots). The scale bar represents 50 km and the arrow indicates a northern orientation. The map was modified from Bisterfeld et al. (2022) using QGIS version 3.22.2 (QGIS Development Team, 2021). The map of Germany was obtained from GeoBasis-DE/BKG (GeoBasis-DE / Bundesamt für Kartographie und Geodäsie, 2022). (For interpretation of the references to colour in this figure legend, the reader is referred to the Web version of this article.)

The hides of 131 wildcats found dead from 2018 to 2020 in the German federal state Rhineland-Palatinate (Fig. 1) were available from the project “Monitoring of dead wildcats in Rhineland-Palatinate (*Totfundmonitoring Wildkatze in Rheinland-Pfalz*)” of the German Federation for the Environment and Nature Conservation, Friends of the Earth Germany, state association of Rhineland-Palatinate (*Bund fuer Umwelt und Naturschutz Deutschland* (*BUND*), *Landesverband Rheinland-Pfalz*). Specimens were dissected by the BUND and the cooperating institutions Will and Liselott Masgeik Foundation, OEKOLOG field research and the Clinic for Birds, Reptiles, Amphibians and Fish, Justus-Liebig-University Giessen (Leonhardt et al., 2021). Parameters such as hybrid status, sex, age, nutritional condition, and state of decomposition were determined during dissections as previously described by Leonhardt et al. (2021). The classification of the nutritional condition was carried out on the basis of subcutaneous, visceral, coronary and kidney fat deposits as described in Bisterfeld et al. (2022), resulting in the categories “very good”, “good”, “moderate”, “bad” and “very bad/cachectic”. The classification of the state of decomposition (“fresh”, “moderate fresh”, “moderate rotten”, “proceeded rotten”) was carried out as part of the BUND project (Leonhardt et al., 2021) as described by Eskens et al. (2016). Skin with fur was collected from the wildcats and stored at −20 °C. According to the recommendations of OIE (International Office of Epizootics, 2008), hides were frozen at −80 °C for at least 48 h before further processing to prevent potential transmission of zoonotic agents, especially *Echinococcus multilocularis*.

### 2.2 Examination of fur and ear canals and morphological identification of ectoparasites

The condition of the skin and fur of each specimen was evaluated regarding missing, wet, bloody, sticky or rotten parts. Subsequently, hides were macroscopically examined for ectoparasites using a flea comb, after we determined that the undercoat was too dense by parting the coat with the thumb or fingers. Cerumen was scraped out of the ear canals and microscopically examined at 100x magnification (Primostar 1; Carl Zeiss Microscopy Deutschland GmbH). If at least one mite was present, additional cerumen was collected from both ear canals and heated in 10% KOH (Carl Roth GmbH + Co. KG, Karlsruhe, Germany) for approximately 1 min until boiling for a few seconds using a microwave. Parasites were counted and microscopically identified to family, genus, or species level by morphological keys (Beck and Prosl, 2010; Biebel, 2007; Boch et al., 2006; Deplazes et al., 2020; Emerson and Price, 1981; Estrada-Peña et al., 2018; Kéler, 1957; Leone et al., 2013; Peus, 1938; Pratt and Wiseman, 1962; Prosl et al., 2004; Smit, 1966; Wall and Shearer, 2008). Due to difficulties in morphologically differentiating of *I. hexagonus* from *I. canisuga* larvae and nymphs, they were summarised as *I. hexagonus*/*canisuga*.

### 2.3 Molecular identification of ectoparasite species

Morphological species identification was confirmed by molecular analysis of at least one specimen of each detected species, except for *T. autumnalis* due to a lack of reference sequences available in National Center for Biotechnology Information (NCBI) GenBank. There was no reference sequence for *F. hercynianus* either, but sequencing was nevertheless carried out to generate the first sequences, and to compare them with available sequences of *F*. *subrostratus*. Due to distinct morphologies, adult ticks were not molecularly identified.

For *I. hexagonus*/*canisuga*, Ceratophyllidae, *Chaetopsylla* spp., and *Ctenophthalmus* spp., molecular analyses were carried out for species differentiation of one to 24 individual specimens, because a morphological species diagnosis was not possible. For DNA extraction, 45 μl DirectPCR Lysis Reagent (DirectPCR® Cell Lysis Reagent; Viagen Biotech, Inc., Los Angeles, CA, USA) and 5 μl Proteinase K (20 mg/ml, MACHEREY-NAGEL GmbH & Co. KG, Dueren, Germany) were used for small specimens (tick larvae, mites, and lice), whereas the volumes were doubled for larger parasites (tick nymphs and fleas). Specimens were disrupted with a pestle and incubated for 5–16 h at 55 °C with subsequent inactivation of Proteinase K at 85 °C for 45 min. Species identification was achieved by amplifying and sequencing the 16S rRNA gene of ticks, the cytochrome *c* oxidase subunit 2 (COX-2) gene and/or the internal transcribed spacer 1 (ITS-1) region of fleas and the cytochrome *c* oxidase subunit 1 (COX-1) gene of *F*. *hercynianus* and *O*. *cynotis.* PCR was performed in a 50 μl reaction mixture containing 0.25 μl (5.0 U/μl) DreamTaq DNA Polymerase, 5.0 μl 10x DreamTaq Buffer (Thermo Fisher Scientific Inc., Waltham, MA, USA) and 1.0 μl dNTPs (10 mM each; Roti®-Mix PCR 3, Carl Roth GmbH + Co. KG, Karlsruhe, Germany). Primers and cycling conditions are listed in detail in Table 1. The primer FeliSeq-R (TCA ACA CAA GAG TTT GCC AAC ACT) for molecular confirmation of *F. hercynianus* was designed based on a COX-1 sequence of *F*. *subrostratus* (GenBank accession no. HM171454) by using the Molecular Biology Application from Benchling® (San Francisco, CA, USA; https://www.benchling.com/molecular-biology). PCR products were subjected to electrophoresis in GelRed®-supplemented (1:10,000; Biotium, Inc., Fremont, CA, USA) 1.5 % agarose gels and custom Sanger sequenced (Microsynth Seqlab GmbH, Goettingen, Germany). Species were determined by BLASTing (basic local alignment search tool) sequences against the NCBI standard databases and subsequently aligned with published reference sequences using Clone Manager (Version 9 Professional, Sci-Ed, Westminster, Colorado, USA). Representative sequences were deposited in GenBank under accession numbers **PP415869**-**PP415870** (*I. ricinus*, 16S rRNA); **PP415859**-**PP415868** (*I. hexagonus*, 16S rRNA); **PP415856**-**PP415858** (*I. canisuga*, 16S rRNA); **PP430732**, **PP430735**, **PP430736**, **PP430738**, **PP430739**, **PP430741**, **PP430742**, **PP430745**, **PP430746**, **PP430748**, **PP430749** (*C. sciurorum*, COX-2); **PP510442** (*C. sciurorum*, ITS-1); **PP430743** (*Nosopsyllus fasciatus*, COX-2); **PP510440** (*Pulex irritans*, ITS-1); **PP510441** (*S. cuniculi*, ITS-1); **PP430733**, **PP430734**, **PP430737** (*Chaetopsylla trichosa*, COX-2); **PP510443** (*C. trichosa*, ITS-1); **PP430747** (*Chaetopsylla globiceps*, COX-2); **PP510444** (*C. felis*, ITS-1); **PP430744** (*Archaeopsylla erinacei*, COX-2); **PP430740** (*Ctenophthalmus* sp., COX-2); **PP510445** (*Ctenophthalmus* sp., ITS-1); **PP425996** (*O*. *cynotis*, COX-1); and **PP425997**-**PP425998** (*F*. *hercynianus*, COX-1).

**Table 1:** PCR identification of detected ectoparasites.

Parasite	Targeted DNA region	Primers	Template/reaction volume	Temperature profile	Amplicon size	Reference(s)
Ticks	16S rRNA	16S + 1, 16S - 1 (0.4 μM each)	0.5–4.0 μl^a^/50 μl	95 °C for 3 min; 7 × 95 °C for 30 s, 47.0–48.8 °C (+0.3 °C each cycle) for 30 s, 72 °C for 45 s; 31 × 95 °C for 30 s, 50 °C for 30 s, 72 °C for 45 s	380 bp	Norris et al. (1996), Mangold et al. (1998), Hauck et al. (2019)
Fleas	COX-2 mt DNA	F-Leu, R-Lys (1.0 μM each)	2.0–5.0 μl/50 μl	95 °C for 3 min; 40 × 95 °C for 30 s, 53 °C for 1 min, 72 °C for 1 min; 72 °C for 7 min	780 bp	Hornok et al. (2018)
	ITS-1	ITS1 sen (1), ITS1 rev (1) (0.4 μM each)	5.0 μl/50 μl	94 °C for 3 min; 35 × 94 °C for 1 min, 54 °C for 1 min, 72 °C for 1 min; 72 °C for 10 min	900-1200 bp	Gamerschlag et al. (2008)
*F*. *hercynianus*	COX-1 mtDNA	L6625, H7005, FeliSeq-R^b^ (1.0 μM each)	5.0 μl/50 μl	95 °C for 2 min; 4 × 95 °C for 1 min, 45 °C for 1 min, 72 °C for 1 min; 30 × 93 °C for 1 min, 60 °C for 1 min, 72 °C for 1 min; 72 °C for 7 min	380 bp	Hafner et al. (1994), this study (FeliSeq-R)
*O*. *cynotis*	COX-1 mtDNA	Fwd, Rev (1.0 μM each)	3.0–10.0 μl/50 μl	95 °C for 3 min; 40 × 95 °C for 30 s, 56 °C for 30 s, 72 °C for 1 min; 72 °C for 5 min	650 bp	Juan et al. (2015)

a Larvae: 4.0 μl, nymphs: 0.5–4.0 μl.

b Both reverse primers H7005 and FeliSeq-R were used for PCR, FeliSeq-R was used for sequencing.

### 2.4 Statistical analyses

Statistical analyses were performed using R version 4.1.0 (R Core Team, 2021). The prevalence of the different stages (adults, nymphs, and larvae) of *I. ricinus* and *I. hexagonus/canisuga* was compared within and between the two tick species by χ^2^ tests, followed by Bonferroni-Holm correction of *P*-values to account for multiple comparisons. To test for negative or positive associations between ectoparasite orders, mathematically expected coinfestation frequencies were calculated by multiplication of the respective prevalence values, which were compared to the observed coinfestation frequencies by χ^2^ tests, or Fisher's Exact tests in case of values ≤ 5, respectively.

Moreover, the influence of the predictor variables ‘season of finding’ and ‘state of decomposition’ on the prevalence of *I. ricinus* and *I. hexagonus*/*canisuga* was calculated using generalised linear models (GLMs) with binominal error structure. Multiple comparisons were performed for the factor ‘season of finding’ using Tukey contrasts with single-step *P*-value adjustment. For both models, a likelihood ratio test was carried out for comparison with a null model that contained only the intercept. Due to missing data for three wildcats, a subset of 128 animals was included in the GLM calculations.

Because calculation of meaningful multivariate models was not possible for the remaining parasites due to low prevalence, seasonal comparisons for total ectoparasites, ticks, fleas, *T. autumnalis*, *O. cynotis*, and *F. hercynianus* were conducted using Fisher's Exact tests in case of values up to five, or χ^2^ tests in case of values above five, respectively, and subsequent Bonferroni-Holm correction of *P*-values.

## 3 Results

### 3.1 Key data of the wildcats

Of 131 wildcats, 48.1% came from Palatinate, 21.4% from Hunsrueck, 12.2% from Westerwald, 10.7% from Eifel, and 3.1% from Taunus (Fig. 1). Two of the animals were suspected to be hybrids between wildcat and domestic cat, the others were morphologically or genetically confirmed as wildcats as previously reported (Bisterfeld et al., 2022; von Thaden et al., 2020). General data of the examined wildcats, e.g. sex, age, nutritional condition, and state of composition are shown in Table 2. Data on sex and/or nutritional status were not available for all cats which were in an advanced state of decomposition or when parts of the carcasses were missing (Leonhardt et al., 2021). There were slightly more males (51.9%) than females (39.7%). Due to advanced decomposition or destruction of the carcasses, sex determination was not possible for the remaining 8.4% of wildcats. The largest sample of wildcats was adults (45.0%). Most animals were in good or very good nutritional condition (57.3%), were mainly found in the autumn months of September, October, and November (43.5%) and almost half of all carcasses were in a moderate fresh state (48.9%). The majority of animals (93.9%) died as a result of trauma, mostly caused by road traffic.

**Table 2:** Key data of the examined wildcat specimens available for the study (n = 131).

Key date	Number of wildcats (%)
Sex	
Male	68 (51.9)
Female	52 (39.7)
Not determinable	11 (8.4)
Age	
Adult (>25 months)	59 (45.0)
Subadult (11–24 months)	23 (17.6)
Immature (5–10 months)	32 (24.4)
Juvenile (<4 months)	10 (7.6)
Not determinable	7 (5.3)
Nutritional condition	
Very good	29 (22.1)
Good	46 (35.1)
Moderate	34 (26.0)
Bad	2 (1.5)
Very bad/cachectic	7 (5.3)
Not determinable	13 (9.9)
Season of finding	
Spring (March, April, May)	29 (22.1)
Summer (June, July, August)	18 (13.7)
Autumn (September, October, November)	57 (43.5)
Winter (December, January, February)	24 (18.3)
Data not available	3 (2.3)
State of decomposition	
Fresh	29 (22.1)
Moderate fresh	64 (48.9)
Moderate rotten	26 (19.8)
Proceeded rotten	12 (9.2)
(Suspected) cause of death	
Trauma	123 (93.9)
Cachexia	1 (0.8)
Unknown	7 (5.3)

### 3.2 Parasites from fur and ear canals

**Fig. 2:**
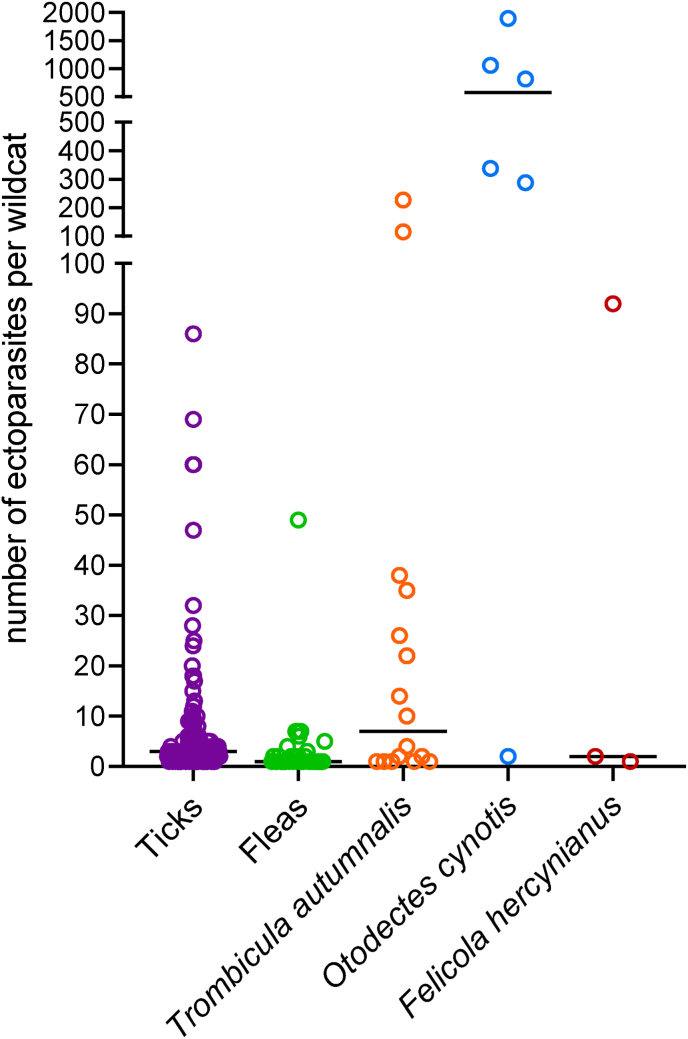
Infestation intensities of *Felis silvestris* from Germany with ticks (n = 95), fleas (n = 36), *Trombicula autumnalis* (n = 16), *Otodectes cynotis* (n = 6), and *Felicola hercynianus* (n = 3). The dots indicate individual values and the lines mark the median. Please note the different scaling in the different sections of the y-axis.

Examination of the entire fur (body and head) was possible for 65 of 131 specimens, whereas 62 hides could only be partially examined due to missing body parts or sticky, bloody or loose fur that did not allow combing. Of four animals, only the head was present. The ear canals of only 124 animals could be examined, as they were completely missing in seven wildcats. Detailed data on ectoparasite prevalence and infestation intensities are shown in Table 3 and Fig. 2.

**Table 3:** Prevalence and infection intensities of ectoparasites in European wildcats from Germany (n = 131).

Order		Positive wildcats	Prevalence (%)	95% CI	Infestation intensity			
					Min.	Max.	Mean	Median
Metastigmata (ticks)	*Ixodes ricinus*	65	49.6	40.8–58.5	1	60	4.8	2.0
	Adults	58	44.3	35.6–53.2	2	60	5.1	2.0
	Females	55	42.0	33.4–50.9	1	48	4.3	2.0
	Males	25	19.1	12.7–26.9	1	12	2.2	1.0
	Nymphs	9	6.9	3.2–12.6	1	2	1.3	1.0
	Larvae	5	3.8	1.3–8.7	1	2	1.2	1.0
	*Ixodes hexagonus*/*canisuga*	48	36.6	28.4–45.5	1	82	9.6	2.0
	Adults	8	6.1	2.7–11.7	1	2	1.1	1.0
	Females (all *I. hexagonus*)	8	6.1	2.7–11.7	1	2	1.1	1.0
	Males	0	0	0	0	0	0	0
	Nymphs	25	19.1	12.7–26.9	1	36	4.6	1.0
	Larvae	33	25.2	18.0–33.5	1	78	10.2	4.0
	*Ixodes* spp.^a^	19	14.5	9.0–21.7	1	5	1.8	1.0
	Adults	1	0.8	0.0–4.2	1	1	1.0	1.0
	Females	1	0.8	0.0–4.2	1	1	1.0	1.0
	Males	0	0	0	0	0	0	0
	Nymphs	1	0.8	0.0–4.2	1	1	1.0	1.0
	Larvae	0	0	0	0	0	0	0
	Total ticks	95	72.5	64.0–80.0	1	86	8.5	3.0
Siphonaptera (fleas)	*Ceratophyllus sciurorum*	16	12.2	7.1–19.1	1	4	1.4	1.0
	*Nosopsyllus fasciatus*	2	1.5	0.2–5.4	1	2	1.5	1.5
	Ceratophyllidae^a^	10	7.6	3.7–13.6	1	3	1.6	1.0
	*Pulex irritans*	7	5.3	2.2–10.7	1	3	1.4	1.0
	*Spilopsyllus cuniculi*	5	3.8	1.3–8.7	1	48	12.0	4.0
	*Chaetopsylla trichosa*	2	1.5	0.2–5.4	1	2	1.5	1.5
	*Chaetopsylla globiceps*	1	0.8	0.0–4.2	1	1	1.0	1.0
	*Chaetopsylla* spp.^a^	1	0.8	0.0–4.2	1	1	1.0	1.0
	*Ctenocephalides felis*	2	1.5	0.2–5.4	1	1	1.0	1.0
	*Archaeopsylla erinacei*	2	1.5	0.2–5.4	1	1	1.0	1.0
	*Ctenophthalmus baeticus*^a^	1	0.8	0.0–4.2	1	1	1.0	1.0
	Total fleas	36	27.5	20.0–36.0	1	49	3.4	1.0
Prostigmata/Astigmata (mites)	*Trombicula autumnalis*	16	12.2	7.1–19.1	1	227	31.3	7.0
	Outer body surface	5	3.8	1.3–8.7	2	111	39.2	26.0
	Ear canals^b^	12	9.7	5.1–16.3	1	227	25.3	3.0
	*Otodectes cynotis*^b^	6	4.8	1.8–10.2	2	1896	733.3	577.0
	Unidentified	11	8.4	4.3–14.5	1	1	1.0	1.0
	Total mites	31	23.7	16.7–31.9	1	1896	158.4	2.0
Phthiraptera (lice)	*Felicola hercynianus*	3	2.3	0.5–6.5	1	92	31.7	2.0
Total ectoparasites		110	84.0	76.5–89.8	1	1896	54.0	4.0

CI: confidence interval.

a (sub)species could not be further differentiated.

b n = 124.

**Fig. 3:**
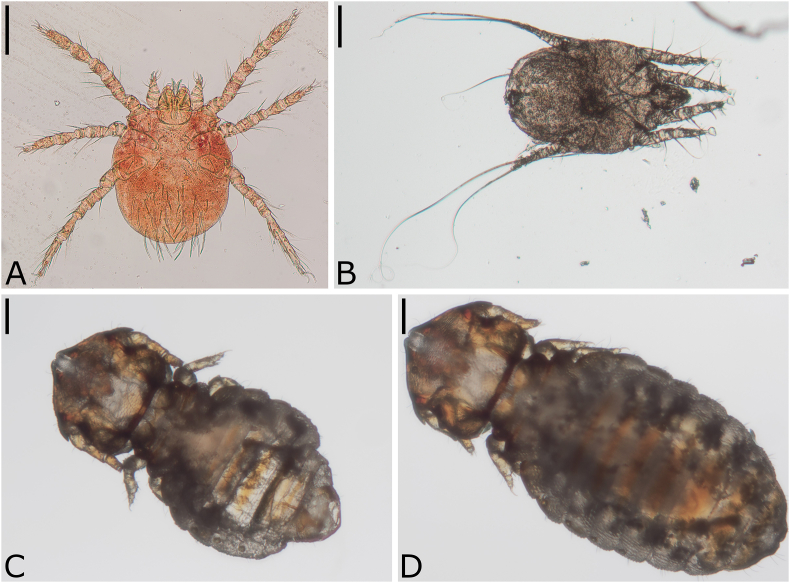
Representative specimens of *Trombicula autumnalis* (A), *Otodectes cynotis* (B), and *Felicola hercynianus* (C-D; C: male, dorsal view, D: female, dorsal view) detected on *Felis silvestris* from Germany. Scale bars represent 100 μm.

**Fig. 4:**
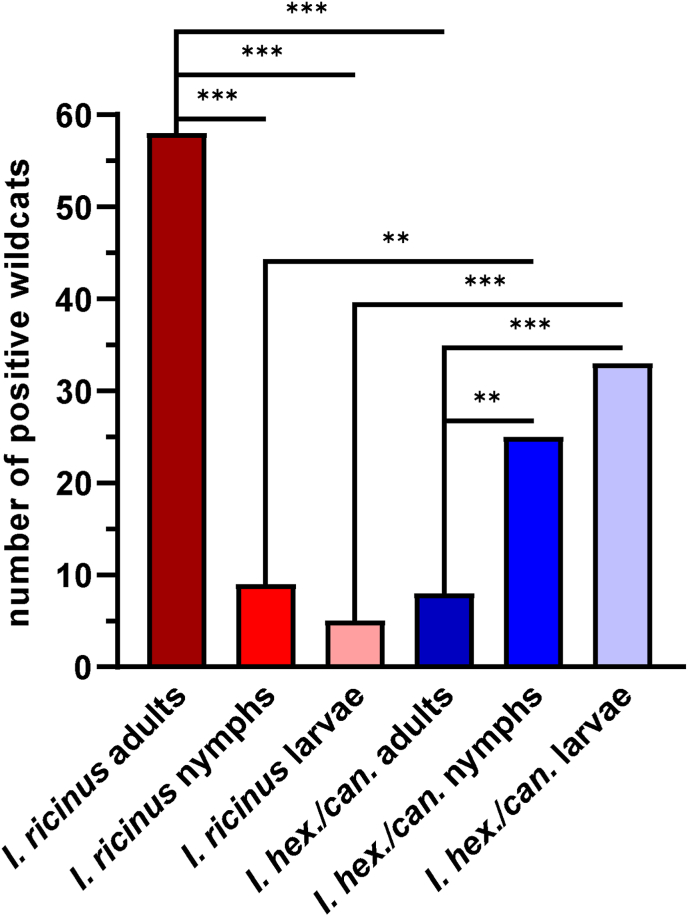
Prevalence of adults, nymphs, and larvae of *Ixodes ricinus* and *Ixodes hexagonus*/*canisuga* on 131 *Felis silvestris* from Germany. Asterisks indicate significant differences after Bonferroni-Holm correction of *P*-values (**P ≤ 0.01 and ***P ≤ 0.001).

In total, 84.0% (110/131) of the wildcats examined were infested with ectoparasites. Ticks were most common (72.5% [95/131]), followed by fleas (27.5% [36/131]), mites (23.7% [31/131]), and lice (2.3% [3/131]; Fig. 3, Fig. S1). Most of the ticks were identified as *I*. *ricinus* (49.6% [65/131]), while *I. hexagonus*/*canisuga* were identified on 36.6% (48/131) of the wildcats. Due to destroyed or incomplete specimens, *Ixodes* ticks from 14.5% (19/131) of the wildcats could only be identified to genus level. The prevalence of adult *I. ricinus* was significantly higher compared to nymphs and larvae of *I. ricinus* (χ^2^ = 46.2, df = 1, *P* < 0.001 [adults vs. nymphs] and χ^2^ = 56.5, df = 1, *P* < 0.001 [adults vs. larvae]) and adult *I. hexagonus/canisuga* (χ^2^ = 48.6, df = 1, *P* < 0.001). In contrast, *I. hexagonus*/*canisuga* nymphs occurred significantly more often than *I. ricinus* nymphs (χ^2^ = 7.6, df = 1, *P* = 0.006) and *I. hexagonus/canisuga* adults (χ^2^ = 8.9, df = 1, *P* = 0.006), while the prevalence of *I. hexagonus/canisuga* larvae was significantly higher compared to *I. ricinus* larvae (χ^2^ = 22.4, df = 1, *P* < 0.001) and *I. hexagonus/canisuga* adults (χ^2^ = 16.7, df = 1, *P* < 0.001; Fig. 4).

A total of nine different flea species were detected. *Ceratophyllus sciurorum* was found on 12.2% (16/131) and *N. fasciatus* on 1.5% (2/131) of the wildcats, whereas 7.6% (10/131) of wildcats harboured Ceratophyllidae that could not be identified to species level. *Chaetopsylla trichosa* was identified on 1.5% (2/131) and *C. globiceps* on 0.8% (1/131) of the wildcats. One wildcat (0.8% [1/131]) was infested with *Chaetopsylla* spp. that could only be differentiated to genus level. Further identified fleas were *P. irritans* (5.3% [7/131]), *S. cuniculi* (3.8% [5/131]), *C. felis* (1.5% [2/131]), *A. erinacei* (1.5% [2/131]), and *C. baeticus* (0.8% [1/131]).

Two mite species, *T. autumnalis*, and *O. cynotis*, were identified on the wildcats. *Trombicula autumnalis* was found on 12.2% [16/131]) of the animals. They were located in the ear canals (75.0 % [12/16]) as well as on the outer body surface (31.3% [5/16]), namely in the Henry's pocket (25.0% [4/16]) and the distal outer surface of the auricles (6.3% [1/16]). Interestingly, *T. autumnalis* was only located on the outer body surface on animals found in September. *Otodectes cynotis* was detected on 4.8% (6/124) of the wildcats and was located exclusively in the ear canals. For 11 wildcats, one mite each could not be identified or rediscovered after KOH processing.

*Felicola hercynianus* was the only louse species detected and was present on 2.3% (3/131) of the animals.

Half (55/110) of the positive animals were mono-infested with ticks, 6.4% (7/110) with fleas, and 5.5% (6/110) with mites. Coinfestations with ticks, fleas, mites, and/or lice were observed in 38.2% (42/110) of the positive wildcats. Ticks and fleas represented the most frequently detected double infestation (14.5% [16/110]), followed by ticks and mites (10.9% [12/110]), fleas and mites (1.8% [2/110]), and ticks and lice (0.9% [1/110]). Triple infestations with ticks, fleas, and mites were observed in nine individuals (8.2%) and two (1.8%) wildcats were infested with ticks, fleas, mites, and lice. Detailed data on ectoparasite coinfestations are shown in Table 4. No significant positive or negative associations between ectoparasite orders were detected, as the observed frequencies did not differ significantly from the mathematically expected frequencies (all *P*-values >0.05).

**Table 4:** Single and coinfestations of ectoparasites in European wildcats from Germany.

Order	Positive wildcats	Prevalence (%) in ectoparasite-infested wildcats (n = 110)	Prevalence (%) [95% CI] in all wildcats (n = 131)	Infestation intensity			
				Min.	Max.	Mean	Median
Ticks only	55	50.0	42.0 [33.4–50.9]	1	69	7.9	2.0
Fleas only	7	6.4	5.3 [2.2–10.7]	1	4	1.6	1.0
Mites only	6	5.5	4.6 [1.7–9.7]	1	1896	456.2	11.5
Ticks + fleas	16	14.5	12.2 [7.1–19.1]	2	52	10.4	6.5
Ticks + mites	12	10.9	9.2 [4.8–15.5]	2	340	50.0	11.5
Ticks + lice	1	0.9	0.8 [0.0–4.2]	152	152	152.0	152.0
Fleas + mites	2	1.8	1.5 [0.2–5.4]	2	289	145.5	145.5
Ticks + fleas + mites	9	8.2	6.9 [3.2–12.6]	5	1064	142.0	17.0
Ticks + fleas + mites + lice	2	1.8	1.5 [0.2–5.4]	33	236	134.5	134.5

CI: confidence interval.

Within orders, coinfestations with *I. ricinus* and *I. hexagonus*/*canisuga* were observed on 21.1% (20/95) of the tick-infested wildcats. Of the flea-infested wildcats, 16.7% (6/36) carried two to three different flea species. Coinfestation with *T. autumnalis* and *O. cynotis* was detected in the ear canals of 3.2% (1/31) of the mite-infested wildcats.

The infestation intensities per wildcat ranged from 1 to 86 for ticks, 1–49 for fleas, 1–227 for *T. autumnalis*, 2–1896 for *O. cynotis*, and 1–92 for *F. hercynianus* (Table 3, Fig. 2). Five *O. cynotis*-infested animals, with infestation intensities of 288–1869 specimens, showed dark brown, coffee ground-like cerumen in their ear canals. In contrast, the ear canals of animals infested with only low numbers of *O. cynotis* (2, one wildcat), with *T. autumnalis* (1–227, 12 wildcats) or those that were negative for ectoparasites had no changes in the appearance of the cerumen.

### 3.3 Molecular species identification

Molecular examination of 1–3 specimens each of *I. ricinus*, *P. irritans*, *S. cuniculi*, *C. felis*, *A. erinacei*, female *C. sciurorum*, female *N. fasciatus*, and *O. cynotis* were performed to confirm morphological differentiation. The 16S rRNA (*I. ricinus*), ITS-1 (*P. irritans*, *S. cuniculi*, *C. felis*), COX-2 (*A. erinacei, C. sciurorum, N. fasciatus*), and COX-1 (*O. cynotis*) sequences were 96.7–100% identical (query cover: 100%) to those of the same species in NCBI GenBank (acc. nos. MN947213, MN947216 [*I. ricinus*, 16S rRNA], EU169198 [*P. irritans*, ITS-1]; EU170157 [*S. cuniculi*, ITS-1]; MT895636 [*C. felis*, ITS-1]; MG637370 [*A. erinacei*, COX-2]; MG637398 [*C. sciurorum*, COX-2]; HQ881597 [*N. fasciatus*, COX-2]; KF891933 [*O. cynotis*, COX-1).

Differentiation between *I. hexagonus* and *I. canisuga*, male *C. sciurorum* and male *N. fasciatus* as well as *C. trichosa* and *C. globiceps* was not feasible based on morphological characteristics. Moreover, morphology did not allow further differentiation of *Ctenophthalmus* species. Therefore, 1–24 specimens of morphologically unidentifiable ectoparasites were analysed molecularly. Of the 58 not differentiable *I. hexagonus*/*canisuga* larvae and nymphs, 24 were identified, of which 21 were *I. hexagonus* and three *I. canisuga*. 16S rRNA sequences showed 100% identity (query cover: 100%) to appropriate reference sequences [acc. nos. KY962076, MK613140 (*I. hexagonus*); KY962075, MK613137 (*I. canisuga*)].

Of the 30 morphologically indistinguishable Ceratophyllidae, genetic differentiation was successful for 14 specimens revealing 13 *C. sciurorum* and one *N. fasciatus*. COX-2 sequences of these species showed 97.6–100% identity (query cover 100%) to respective reference sequences (acc. no. MG637398 [*C. sciurorum*, COX-2]; MG637390 [*N. fasciatus*, COX-2]). Molecular identification failed for 11 specimens and was not performed for five specimens because other Ceratophyllidae of the respective wildcat had already been identified. Of the four COX-2 sequences of *Chaetopsylla* spp., three sequences revealed 99.9–100% identity (query cover 100%) with that of *C. trichosa* (acc. no. MG637393) and one sequence showed 99.7% identity (query cover 100%) with that of *C. globiceps* (acc. no. MG637375). Molecular examination of one *Chaetopsylla* spp. was not successful. The only detected *Ctenophthalmus* spp. was identified as *C*. *baeticus* ssp. based on its COX-2 sequence, which was 98.7% identical (query cover: 100%) to a *C*. *baeticus arvernus* (acc. no. LR991713) as well as a *C*. *baeticus boisseauorum* sequence (acc. no. LR991708).

Due to the absence of appropriate reference sequences for *T. autumnalis* and *F. hercynianus*, molecular confirmation of morphological differentiation was not possible for these species. Nevertheless, the *F. hercynianus* COX-1 sequences showed 73.3–74.1% identity (query cover: 77–91%) to *F. subrostrarus* sequences (acc. nos. OQ682409-OQ682415, AF545700, HM171454).

### 3.4 Seasonal patterns of ectoparasite infestations

**Fig. 5:**
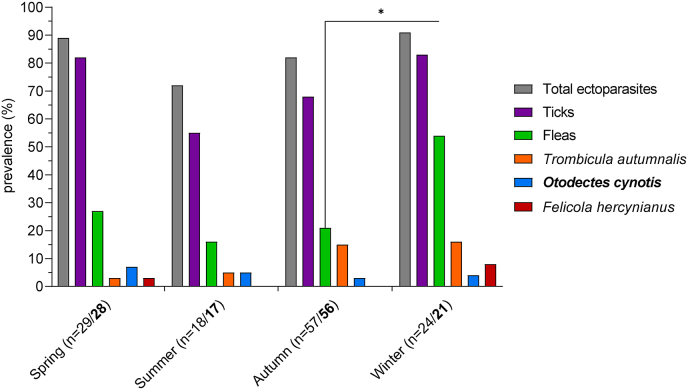
Total ectoparasite prevalence as well as prevalence of ticks, fleas, *Trombicula autumnalis*, *Otodectes cynotis*, and *Felicola hercynianus* on *Felis silvestris* from Germany for each season. Values for *O. cynotis* are printed in bold. The asterisk indicates a significant difference after Bonferroni-Holm correction of *P*-values (*P ≤ 0.05).

Ectoparasites were detected throughout the whole year with slightly higher prevalence values in spring (March, April, and May; 89.7% [26/29]) and winter (December, January, and February; 91.7% [22/24]) than in summer (June, July, and August; 72.2% [13/18]) and autumn (September, October, and November; 82.5% [47/57]) (Fig. 5). Ticks tended to be more prevalent in spring (82.8% [24/29]) and winter (83.3% [20/24]) than in summer (55.6% [10/18]) and autumn (68.4% [39/57]). Regarding the different tick species, statistically significant seasonal differences were detected for *I. ricinus*, with a significantly higher prevalence in spring (65.5% [19/29]; *P* = 0.032) and in winter (66.7% [16/24]; *P* = 0.020) than in autumn (33.3% [19/57]; Table 5). No significant influence of the state of decomposition was found for *I. ricinus*. For *I. hexagonus*/*canisuga*, no statistically significant influence of season nor state of decomposition was detected as the GLM did not differ significantly from the corresponding null model (χ^2^ = 4.6, df = 5, *P* = 0.466; Table 5).

**Table 5:** Results of GLMs testing the influence of different predictor variables on the prevalence of *Ixodes ricinus* and *Ixodes hexagonus/canisuga*.

	*I. ricinus*				*I. hexagonus*/*canisuga*			
	Estimate	SE	*z*	*P*	Estimate	SE	*z*	*P*
Intercept	0.194	0.518	0.374	0.709	0.007	0.511	0.013	0.989
Season of finding								
summer vs. spring	−0.409	0.623	−0.656	0.912	−0.499	0.658	−0.758	0.871
autumn vs. spring	−1.340	0.493	−2.719	0.032^a^	0.166	0.481	0.345	0.986
winter vs. spring	0.191	0.596	0.321	0.988	−0.680	0.625	−1.087	0.692
autumn vs. summer	−0.931	0.566	−1.646	0.349	0.664	0.606	1.096	0.687
winter vs. summer	0.600	0.657	0.913	0.795	−0.181	0.721	−0.251	0.994
winter vs. autumn	1.531	0.532	2.881	0.020^a^	−0.845	0.560	−1.510	0.425
State of decomposition (ref. fresh)								
moderate fresh/moderate rotten	0.475	0.463	1.025	0.305	−0.676	0.455	−1.486	0.137
proceeded rotten	1.100	0.761	1.444	0.149	−0.642	0.738	−0.870	0.384

SE: standard error.

The full model of *I. ricinus* was significantly different from a null model containing only the intercept (χ2 = 14.6, df = 5, *P* = 0.012).

The full model of *I. hexagonus*/*canisuga* was not significantly different from a null model containing only the intercept (χ^2^ = 4.6, df = 5, *P* = 0.466).

a Significant *P*-values (≤0.05).

The prevalence of fleas was highest in winter (54.2% [13/24]) and lower during the remainder of the year (spring 27.6% [8/29], summer 16.7% [3/18], autumn 21.1% [12/57]), with a statistically significant difference between winter and autumn (χ^2^ = 7.2, df = 1, *P* = 0.044).

For the remaining ectoparasites, no statistically significant seasonal differences were found. Seasonal infestation rates with *T. autumnalis* amounted to 3.4% in spring (1/29), 5.6% in summer (1/18), 21.1% in autumn (12/57), and 16.7% in winter (4/24). The prevalence of *O. cynotis* was similar during all seasons (spring 7.1% [2/28], summer 5.9% [1/17], autumn 3.6% [2/56], winter 4.8% [1/21]) and *F. hercynianus* was only found in spring (3.4% [1/29]) and winter (8.3% [2/24]).

## 4 Discussion

Although collection of data on ectoparasite infestation from dead animals has proven to be difficult, 15 different ectoparasite species were detected in this study on German wildcats. Studies on ectoparasite infestation of dead found animals are rarely performed due to the fact that ectoparasites can leave their dead host (Leple, 2001; Lledo et al., 2015) and that the sample quality can be poor due to decay, destroyed, wet/bloody carcasses, or missing body parts. While there is comparatively many data available on endoparasites of wildcats from different European countries, e.g. Greece, Croatia, Germany, Romania, and Italy (Deak et al., 2022; Diakou et al., 2021; Krone et al., 2008; Martinković et al., 2017; Napoli et al., 2016; Steeb, 2015), fewer and less recent studies on ectoparasites, mostly limited to Slovenia, Spain, and France, have been published (Brglez and Železnik, 1976; Dominguez, 2004; Leple, 2001). To the best of our knowledge, this is the first study to determine the prevalence of ectoparasites on a large sample size of more than 130 wildcats in Germany.

Most ectoparasite species of wildcats could be identified successfully by morphological characteristics or genetic analyses. Although two genes (ITS-1 and COX-2) of the discovered *C. baeticus* specimen were amplified and numerous reference sequences of *Ctenophthalmus* spp. are available in GenBank, the subspecies could not be conclusively determined. As also reported by others, the ITS-1 region is not suitable for *Ctenophthalmus* species identification (Zurita and Cutillas, 2021) and the COX-2 sequence matched both *C. baeticus arvernus* and *C. baeticus boisseauorum*. Hence, the specimen is therefore most likely *C. baeticus*, but the subspecies could not be determined.

Unfortunately, there were no reference sequences for *F. hercynianus* available in NCBI GenBank. Nevertheless, the lice could be identified with certainty, as *F. hercynianus* can be easily delineated from other louse species that occur on small felids in Europe by its well described morphological characteristics (Kéler, 1957; Martín Mateo, 2009; Mey, 1988). Nevertheless, its COX-1 gene was sequenced so that the database could be supplemented.

In this study, ticks were found on 72.5% of the wildcats with *I*. *ricinus* being more prevalent (49.6%) than *I. hexagonus*/*canisuga* (36.6%). In France, the overall prevalence of ticks was lower with eight of 39 (20.5%) *Ixodes* spp.-infested wildcats (Leple, 2001). The distribution pattern of *I. ricinus* stages showed a significantly higher frequency of adult than immature ticks. This is in accordance with a study from Thuringia, where 1286 fox carcasses were examined for ticks. The majority of adult ticks (82.2% [3711/4513]) was identified as *I. ricinus* while adult *I. canisuga* (10.8% [486/4513]) and *I. hexagonus* (6.7% [303/4513]) were less common (Meyer-Kayser et al., 2012). Immature stages of *I. hexagonus*/*canisuga*, which were summarised due to difficulties in the morphological differentiation of larvae and nymphs, were significantly more common on wildcats than adult ticks, although a stage-dependent difference in host preference is rarely described for these species (Arthur, 1953; Estrada-Peña et al., 2018; Hillyard, 1996). In the fox study, 71.7% (2001/2790) of the *I. canisuga* and *I. hexagonus* were nymphs and only 28.3% (789/2790) were adults (Meyer-Kayser et al., 2012). An explanation might be that *I. hexagonus* and *I. canisuga* are nest-dwelling ticks with all stages parasitising the same host (Estrada-Peña et al., 2018). Since the population density of immature stages is higher than that of adult ticks, a higher infestation rate with immature stages is likely.

Fleas were detected less frequently (27.5%) than ticks, presumably because they are more mobile and can therefore quickly leave a dead host (Hsu and Wu, 2001). In Spain, the flea species *C. sciurorum*, *S. cuniculi*, *C. felis*, *Ctenophthalmus* spp., and *P. s. spectabilis,* were detected on four of six (66.7%) wildcats (Dominguez, 2004). With nine different flea species, the diversity of fleas in the present study was considerably higher. Many of these fleas are associated with prey species of wildcats or can be present in burrows of these prey animals. *Ceratophyllus sciurorum* mainly parasitises red squirrels (*Sciurus vulgaris*), but also small rodents such as dormice. Besides humans, *P. irritans* prefers animals that live in large burrows such as badgers and foxes. Rabbits are the main hosts of *S. cuniculi,* but they can also be found on domestic cats. *Chaetopsylla globiceps* infest mainly foxes, while *C. trichosa* is found on both foxes and badgers. The main hosts for the other flea species that were found, namely *N. fasciatus*, *C. felis*, *A. erinacei*, and *C. baeticus*, are rats (*Rattus* spp.), domestic cats, hedgehogs, and rodents, respectively (Smit, 1966). Wildcats use e.g. hollow tree trunks and brushwood piles, but also fox and badger burrows as nursery sites (Piechocki, 1990). In Germany they prey mainly on small rodents and less frequently on hares and shrews (Soricidae) (Lang, 2016). Accordingly, this lifestyle creates interfaces of wildcats with many of the fleas' main hosts or their nests, making transmission to the wildcat likely. Piechocki (1990) mentioned the ‘cat flea’ *C. felis* as a common parasite of wildcats. However, in the present study, only two specimens were detected on one wildcat each. This is probably caused by the fact that *C. felis* is highly temperature-sensitive with most rapid development in warm indoor environments (Deplazes et al., 2020).

*Trombicula autumnalis* was detected on more than one-tenth (12.2%) of the wildcats. Since the ear canals were absent in seven animals, this prevalence might be slightly underestimated. *Trombicula autumnalis* is the most common chigger mite in Europe, occurring on mammals, including humans, birds, and reptiles (Bowman et al., 2002; Giannoulopoulos et al., 2012; Scholer et al., 2006). In the wildcats, *T. autumnalis* was predominantly located in the ear canals, but the Henry's pocket was also frequently infested. In domestic cats, *T*. *autumnalis* is also often located in the Henry's pocket, but also infests other areas of the head such as the chin, temples, lips, eyelids, and the body, e.g. interdigital areas, abdomen as well as the perianal region (Bowman et al., 2002; Leone et al., 2013). However, *T. autumnalis* was only found on the head of the wildcats, possibly due to the long and dense fur on the rest of the body that hampered examination. *Otodectes cynotis* was detected less frequently than *T. autumnalis*, namely on 4.8% of the wildcats. The ear mite frequently infests pets, thus being the most common cause of otitis externa in domestic animals (Bowman et al., 2002; Fanelli et al., 2020).

The wildcats’ chewing louse *F. hercynianus* was detected on three (2.3%) wildcats. In Slovenia, the prevalence of Mallophaga was higher with two of 12 (16.6%) wildcats being infested (Brglez and Železnik, 1976).

Over one third (38.2% [42/110]) of all positive wildcats were infested with at least two different types of ectoparasites, e.g. with ticks and fleas in 16 (14.5%) cases followed by ticks and mites in 12 (10.9%) cases. Coinfestations with ticks, fleas, and lice were relatively common (8.2% [9/110]), while those with ticks and lice (0.9% [1/110]), fleas and mites, or ticks, fleas, mites and lice (1.8% [2/110] each) were detected only occasionally. All lice infestations were coinfestations with other ectoparasites. In Spain, a total of 105 wild animals, including six wildcats, 26 foxes, 22 hedgehogs, and seven squirrels, were examined for ectoparasites, with various species of ticks, fleas, and lice being detected. Of all 87 positive animals, 37 (42.5%) were mono-infested, while 50 (57.5%) were coinfested with up to four different ectoparasite species (Dominguez, 2004).

*Ixodes ricinus* occurred more frequently in spring and winter than in autumn. In contrast, on foxes from Thuringia, *I. ricinus* nymphs and adults were detected significantly more frequently in the warm (April to September) than in the cold (October to March) months (Meyer-Kayser et al., 2012). Interestingly, the highest infestation frequencies of *I. ricinus* and of ticks in general were noted on wildcats collected in winter (66.7% and 83.3%, respectively) and spring (65.5% and 82.8%, respectively), whereas activity of ticks is reported to be rather low in winter (Gethmann et al., 2020; Hagedorn, 2013; Schulz et al., 2014). Fleas were also detected most frequently in winter (54.2%) and significantly less frequently in autumn (21.1%). Possibly, ticks and fleas had lower movement activity due to cold temperatures during the winter months and were therefore not able to leave their dead host. Moreover, the carcasses were better preserved in winter with only 8.3% (2/24) being in a moderately rotten state. Therefore, the ticks and fleas might have been easier to find.

*Trombicula autumnalis* mites are only parasitic during their larval stage. They occur seasonally with high abundance in late summer and autumn (Wall and Shearer, 2008). Accordingly, 62.5% of the 16 wildcats infested with *T. autumnalis* were found in autumn in the present study but seasonal differences were not statistically significant. Infestations of domestic cats with these mites occur mainly in autumn, but cases during the rest of the year are also described (Leone et al., 2013). For *O. cynotis* and *F. hercynianus*, no seasonal patterns in infestation frequency were detected.

Besides prevalence, the infestation intensity is an important parameter in the assessment of ectoparasite infestation because high intensities can have detrimental effects on wildlife population health. In general, most of the wildcats were only mildly infested with few specimens of each parasite species, although high infestation intensities were noted in single individuals. Moreover, the majority of the animals were in good or very good nutritional condition, indicating that the wildcat population appears to be generally healthy.

Low infestation intensities in this and also other studies of wildlife populations are likely related to the fact that dead found animals were examined. Ectoparasites can leave their dead host, so intensities on living wildcats are likely higher. Hence, our intensities should be treated with caution. Because comparative data are not available and no further examinations could be performed, relating infestation intensities or coinfestations to the health status of the wildcats is difficult. Nevertheless, in the present study, infestation intensities on some of the wildcats were elevated compared to those of other specimens. For instance, one wildcat was infested with 86 ticks. On another wildcat, 48 *S. cuniculi* were detected, whereas the intensity of other flea infestations was limited to few (1–7) specimens. The observed *S. cuniculi* infestation intensities, combined with the frequent observation of *S. cuniculi* on wildcats (Dominguez, 2004; Piechocki, 1990; Steeb, 2015), suggests that wildcats are suitable hosts for the rabbit flea, whereas other less common fleas such as *N. fasciatus* and *C. baeticus* probably represent incidental infestations via prey. Both ticks and fleas may cause anaemia in young or weakened animals in heavy infestations (Anderson, 2000; Deplazes et al., 2020).

Unfortunately, comparative data on infestation intensities of mite- and lice-infested wildcats do not exist. The infestation intensity with *O. cynotis* was highly variable with 2–1896 mites found per wildcat. Regarding domestic cats, the intensity of infestation is similarly variable with one case reporting 8500 mites in a single ear (Bowman et al., 2002; Preisler, 1985). *Otodectes cynotis* are distributed worldwide, infest various carnivores such as cats, lynx, foxes, and martens and can cause ear mange, otitis externa, itching, and head shaking (Deplazes et al., 2020; Sotiraki et al., 2001; Wall and Shearer, 2008). In the present study, it was noticeable that increased and coffee ground-like dark cerumen only appeared in the five wildcats infested with 288 or more specimens of *O. cynotis* but was absent in the wildcat with only two detected *O. cynotis* mites or those carrying *T. autumnalis*. However, the number of only six cases does not provide representative results. Furthermore, at least in domestic cats, the amount of discharge does not determine the clinical signs (Sotiraki et al., 2001), so no clinical consequences of *O. cynotis* infestation can be inferred from the data collected in this study.

Larval stages of *T. autumnalis* are not host specific and infest e.g. small mammals and pets such as cats and dogs. Typical symptoms caused by infestation with *T. autumnalis* are itching, pruritus and, as detected in the present study, orange crumbs on the skin, but in many cases, infestations are also asymptomatic (Deplazes et al., 2020; Leone et al., 2013; Wall and Shearer, 2008).

Concerning lice, pediculosis in cats normally does not affect healthy animals. In debilitated or older animals with diminished grooming behaviour, the number of chewing lice may increase, resulting in possible clinical signs such as itching, dull, scaly fur, and alopecia (Bowman et al., 2002; Deplazes et al., 2020; Wall and Shearer, 2008). The at least 48-month-old wildcat infested with 92 *F. hercynianus* in the present study was in a cachectic nutritional condition, suggesting that the lice were able to multiply on the weakened cat.

Because ectoparasites on wildcats can also be harmful to domestic animals, the question arises as to how high the probability may be for transmission from wild to domestic animals and humans. The risk of infestation of domestic cats with *F. hercynianus* is negligible, because *F. hercynianus* seems to solely infest wildcats, whereas domestic cats are usually parasitised by *F*. *subrostratus* (Bowman et al., 2002; Deplazes et al., 2020; Kéler, 1957; Wall and Shearer, 2008). Flea species that commonly infest wildcats, e.g. *C. sciurorum*, *P. irritans*, and *S. cuniculi* are rare on domestic cats and *vice versa* (Deplazes et al., 2020; Visser et al., 2001). Transmission of *O. cynotis* requires direct contact (Wall and Shearer, 2008) but low rates of hybridisation (3–5%) between wild and domestic cats in Germany (Tiesmeyer et al., 2020) indicate that direct contact between the two species is rather rare, wherefore transmission of *O. cynotis* to domestic cats seems to be negligible.

## 5 Conclusions

A broad spectrum of 15 different ectoparasite species, namely three tick, nine flea, two mite, and one chewing louse species, were present on more than 80% of the wildcats. While more than two-thirds of the wildcats harboured ticks and about one-fourth showed infestations with fleas and mites, chewing lice were only occasionally detected. Although the influence of parasites on the health of dead found hosts is difficult to assess, the studied wildcat population seems to be healthy based on their mostly good or very good nutritional condition, whereby ectoparasite infestations appear to play only a minor role in the overall health of the German wildcat population. Nevertheless, individual animals, especially those that are young, old, or weakened, may be harmed by direct or indirect mechanisms, such as blood loss, skin irritation, and transmission of vector-borne diseases.

## Funding

This work was financially supported by the Rhineland-Palatinate Ministry of Environment, Energy, Food and Forestry [*Ministerium für Umwelt, Energie, Ernährung und Forsten Rheinland-Pfalz* (*MUEEF*)]; executed by the German Federation for the Environment and 10.13039/501100020487Nature Conservation, Friends of the Earth Germany, state association of Rhineland-Palatinate [*Bund fuer Umwelt und Naturschutz Deutschland* (*BUND*), *Landesverband Rheinland-Pfalz*] as project management organisation. We acknowledge financial support by the Open Access Publication Fund of the University of Veterinary Medicine Hannover, Foundation.

## Ethics approval and consent to participate

The material examined in this study originated exclusively from wildcats found dead. The analysis was conducted as part of the project “Monitoring of dead wildcats in Rhineland-Palatinate (*Totfundmonitoring Wildkatze in Rheinland-Pfalz*)” of the German Federation for the Environment and Nature Conservation, Friends of the Earth Germany, state association of Rhineland-Palatinate [*Bund fuer Umwelt und Naturschutz Deutschland* (*BUND*), *Landesverband Rheinland-Pfalz*] and was authorised by the responsible Nature Conservation Agencies (Leonhardt et al., 2021).

## Competing interests

The authors declare that they have no competing interests.

## Note

Supplementary data associated with this article.

## CRediT authorship contribution statement

**Katrin Bisterfeld:** Writing – review & editing, Writing – original draft, Visualization, Validation, Investigation, Formal analysis, Data curation. **Marie-Kristin Raulf:** Writing – review & editing, Visualization, Validation, Supervision, Formal analysis, Data curation. **Andrea Springer:** Writing – review & editing, Visualization, Validation, Methodology, Data curation. **Johannes Lang:** Writing – review & editing, Validation, Methodology, Investigation. **Michael Lierz:** Writing – review & editing, Validation, Investigation, Conceptualization. **Christina Strube:** Writing – review & editing, Validation, Supervision, Software, Resources, Methodology, Investigation, Formal analysis, Data curation, Conceptualization. **Ursula Siebert:** Writing – review & editing, Validation, Supervision, Project administration, Investigation, Funding acquisition, Data curation, Conceptualization.

## Declaration of competing interest

The authors declare that they have no known competing financial interests or personal relationships that could have appeared to influence the work reported in this paper.
