# Supplementary material for: Ectoparasites of the European wildcat (Felis silvestris) in Germany
Source: Int J Parasitol Parasites Wildl. 2024 Aug 24;25:100977. doi: 10.1016/j.ijppaw.2024.100977 (PMC11407961; doi:10.1016/j.ijppaw.2024.100977)
Supplement: Multimedia component 1 [file mmc1.docx]

**Table S1** Detection frequencies of Ixodes ricinus and Ixodes hexagonus/canisuga for each predictor variable.

|  |  | ***Ixodes ricinus*** | | ***Ixodes hexagonus/canisuga*** | |
| --- | --- | --- | --- | --- | --- |
| Predictor variable | Total wildcats | Positives | Prevalence (%) | Positives | Prevalence (%) |
| Season of finding |  |  |  |  |  |
| spring | 29 | 19 | 65.5 | 11 | 37.9 |
| summer | 18 | 10 | 55.6 | 5 | 27.8 |
| autumn | 57 | 19 | 33.3 | 23 | 40.4 |
| winter | 24 | 16 | 66.7 | 6 | 25.0 |
| State of decomposition |  |  |  |  |  |
| fresh | 29 | 13 | 44.8 | 13 | 44.8 |
| moderate fresh/moderate rotten | 87 | 43 | 49.4 | 28 | 32.2 |
| proceeded rotten | 12 | 8 | 66.7 | 4 | 33.3 |
