# Supplementary material for: Ectoparasites of the European wildcat (Felis silvestris) in Germany
Source: Int J Parasitol Parasites Wildl. 2024 Aug 24;25:100977. doi: 10.1016/j.ijppaw.2024.100977 (PMC11407961; doi:10.1016/j.ijppaw.2024.100977)
Supplement: Multimedia component 2 [file mmc2.docx]

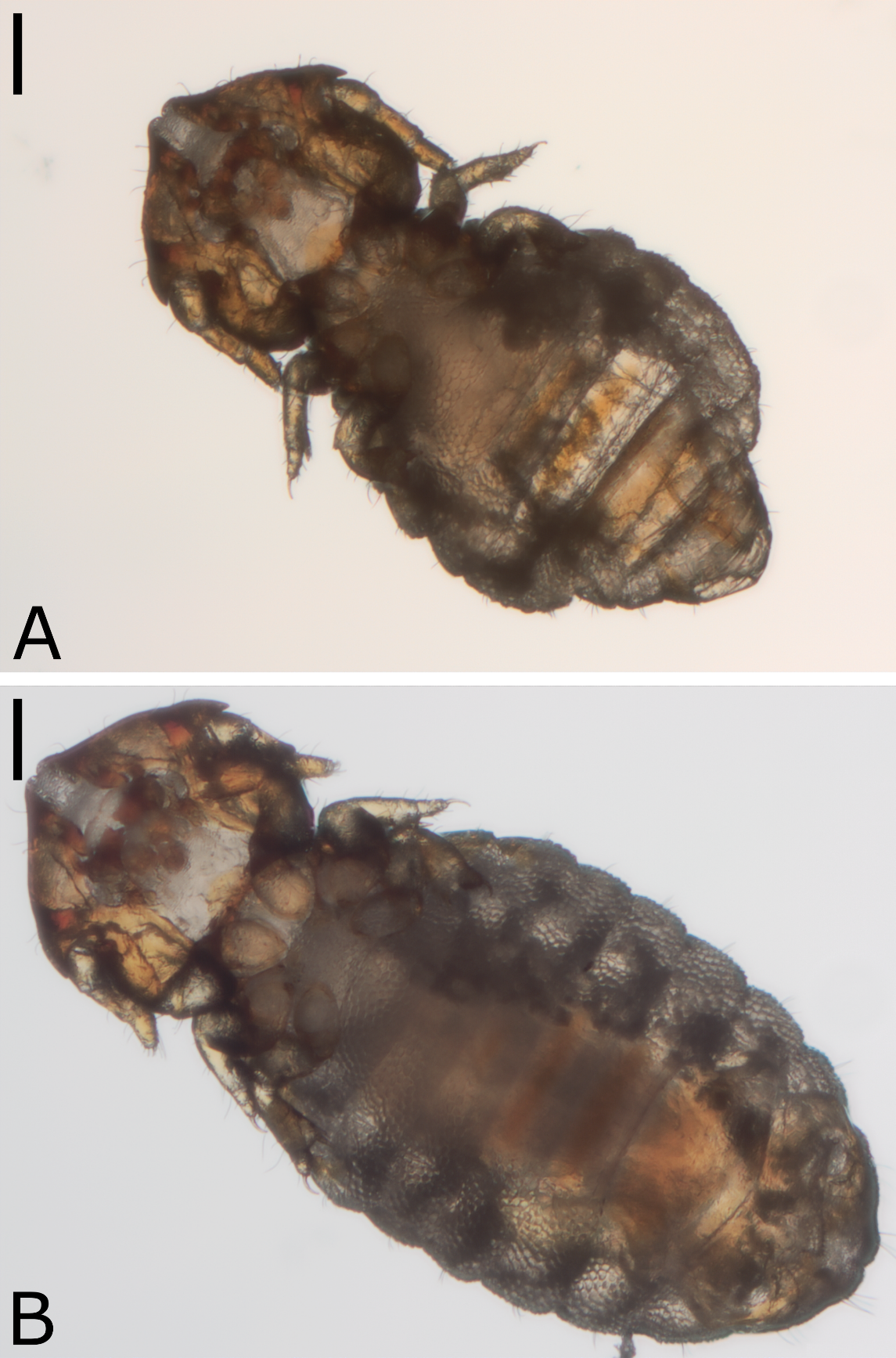


**Figure S1** Ventral view of male (A) and female (B) specimen of Felicola hercynianus. Scale bars represent 100 µm.
